# Bibliography

**Published:** 1894-07

**Authors:** 


### Bibliography.

THE DISCOVERY OF MODERN ANÆSTHESIA. BY WHOM WAS IT MADE? A BRIEF STATEMENT OF FACTS. By Dr. Laird W. Nevius, Specialist, New York.

This book seems to be another effort to solve a problem already settled in the minds of intelligent readers,—that of the discovery of anæsthesia. The author has undertaken to "compile in one small volume undeniable facts and figures . . . by what means or under what circumstances each of the four aspirants performed their first operations upon the basis of which they claimed the honor of being the original discoverer."

It is very evident that the author, while he claims impartiality, entertains the opinion that Dr. Crawford W. Long, of Georgia, is entitled to the credit of discovering anæsthesia, for he is placed first in the series of biographical chapters. While this gentleman claims to have used ether in 1842, and that he first saw it noticed in the *Medical Examiner* for December, 1846; he "was prevented by a very laborious country practice" from communicating his discovery "until the *Medical Examiner* for January, 1847, was received. . . . On reading these articles I determined to wait a few months before publishing an account of my discovery." This probably brings it to near the close of 1848, or six years after the alleged discovery, and four years after Wells had proved the value of nitrous oxide as an anæsthetic. It is quite safe to dismiss Dr. Long as one of those discoverers too late for recognition.

The book contains excellent illustrations of Drs. Long, Wells, Riggs, Jackson, and Morton; the monument to Dr. Wells erected in Hartford; Ether Monument, Boston; bust of Sir James Simpson, and full length illustration of this discoverer of chloroform, and also Dr. Colton.

The book is presented in a very satisfactory manner as far as general make-up is concerned, but the insertion of the full length illustration of the author as a frontispiece, and his office on another page, with himself and assistant at work, is in such exceeding bad taste that it will tend to destroy what might have otherwise been a valuable contribution to the literature of the subject.

